# Timing and Patterns in the Taxonomic Diversification of Lepidoptera (Butterflies and Moths)

**DOI:** 10.1371/journal.pone.0080875

**Published:** 2013-11-25

**Authors:** Niklas Wahlberg, Christopher W. Wheat, Carlos Peña

**Affiliations:** 1 Department of Biology, University of Turku, Turku, Finland; 2 Department of Zoology, Stockholm University, Stockholm, Sweden; BiK-F Biodiversity and Climate Research Center, Germany

## Abstract

The macroevolutionary history of the megadiverse insect order Lepidoptera remains little-known, yet coevolutionary dynamics with their angiospermous host plants are thought to have influenced their diversification significantly. We estimate the divergence times of all higher-level lineages of Lepidoptera, including most extant families. We find that the diversification of major lineages in Lepidoptera are approximately equal in age to the crown group of angiosperms and that there appear to have been three significant increases in diversification rates among Lepidoptera over evolutionary time: 1) at the origin of the crown group of Ditrysia about 150 million years ago (mya), 2) at the origin of the stem group of Apoditrysia about 120 mya and finally 3) a spectacular increase at the origin of the stem group of the quadrifid noctuoids about 70 mya. In addition, there appears to be a significant increase in diversification rate in multiple lineages around 90 mya, which is concordant with the radiation of angiosperms. Almost all extant families appear to have begun diversifying soon after the Cretaceous/Paleogene event 65.51 mya.

## Introduction

Lepidoptera is the second largest order after Coleoptera of largely phytophagous insects, and the largest order that is almost entirely associated with angiospermous plants [1]. The order comprises 157,424 described species [2], and a likely similar number of undescribed species. Given their popularity with amateur and professional entomologists, the life histories and geographic distributions of Lepidoptera are arguably the best known among Insecta [3]. The intricate relationships of Lepidoptera with their host plants have long provided evolutionary biologists a much needed empirical foundation for investigating coevolutionary dynamics [4], [5]. Nevertheless, the factors leading to their evolutionary success are still unclear, as their evolutionary history remains uncertain at two fundamental levels: 1) the phylogenetic relationships of most superfamilies has been unclear and consequently 2) the timing of the major diversification events is unknown.

The phylogenetic relationships of the major clades of Lepidoptera have only recently been studied in detail, beginning with the landmark publication by Kristensen [6], based upon morphology, which was followed by two independent molecular studies [7]–[9]. Common to all of these studies are fairly well-supported clades that can be defined as superfamilies, but very poor support for relationships among the superfamilies. This is most apparent in Ditrysia, which contains 99% of all lepidopteran species. Particularly striking is the preponderance of extremely short internal branches leading to the superfamily clades in the molecular studies [7]–[9]. Here we ask whether this short branching is an artifact of too little data, or the likely result of rapid adaptive radiations associated with the rise of angiosperms, as postulated by Ehrlich and Raven [4] in their untested hypothesis of coevolutionary dynamics.

The timing of the major Lepidoptera diversifications would shed light on the relationship between these phytophagous insects and their larval hosts, the angiosperms. Whether angiosperms were colonized only once by an ancestral lineage is not clear, for a number of extant lineages could have switched to angiosperms independently once they became available as a resource [10]. The lack of robust estimates of when the major lineages have diverged from each other leads to this uncertainty in these early insect plant interactions. Lepidoptera are unfortunately characterized by a lack of fossils that can be confidently assigned to extant clades [11]. The oldest fossil that can be confidently assigned to Lepidoptera is from the Early Jurassic (ca. 190 million years ago, mya). The sister clade of Lepidoptera, Trichoptera, also has a robust fossil of similar age, suggesting that both groups were extant in the beginning of the Jurassic age some 200 mya [12]. Based on the phylogenetic hypothesis of Kristensen [6] and their fossil placements, Grimaldi and Engel [11] suggest that the crown clade of Lepidoptera began diversifying in the late Jurassic (ca. 155 mya), with the main diversification events of the ditrysian lineages happening in the late Cretaceous and Paleocene (between 100 and 55 mya). This hypothesis of late diversification of the Ditrysia necessarily implies that they colonized already diversified angiosperms, effectively removing coevolutionary interactions as a driving factor in the evolutionary origins of these insects and their host plants' early evolutionary history.

However, these late diversification dates have recently been challenged by studies using Bayesian relaxed clock methods and molecular data for various subgroups of Lepidoptera, especially for butterflies [13]–[16]. These studies have found that the age of Papilionoidea is roughly 40 million years older than suggested by the fossil record, with the lineages leading to families diverging from each other in the mid Cretaceous about 100 mya. Importantly, these divergences are much closer in time to the origins of the major angiosperm clades. Consequently, other lineages in Lepidoptera must be older to accommodate for this, but there have been no explicit analyses of these implications. Recently, phylogenomic analysis of the times of divergence in Arthropoda, which included several lineages of Lepidoptera, suggests that a nonditrysian lineage (Prodoxidae) diverged from the ditrysian lineages in the early Jurassic [17]. Molecular evidence is thus pointing to an older Lepidoptera than was earlier believed, potentially suggesting a significant coevolutionary role in the origins of this order.

Here we use the phylogenetic hypothesis and molecular data from Mutanen *et al*. [8] to estimate times of divergence of the major lineages of Lepidoptera using a Bayesian relaxed clock method [18]. The robustness of our findings was assessed by analyzing datasets of smaller and larger sizes than our observed data, finding consistent support for rapid radiations during the rise of the angiosperms, in line with previous untested hypotheses about the role of coevolutionary dynamics in generating species diversity in Lepidoptera.

## Materials and Methods

Analyses were based on the data and the maximum likelihood topology of Mutanen *et al*. [8], which was based on DNA sequences of eight protein coding genes from 350 taxa sampled across all of Lepidoptera. The molecular data did not include the third codon positions for all the gene regions except *elongation factor 1 alpha*. The program BEAST v1.7 [19] was used to estimate times of divergence, with calibrations using seven nodes (Table 1), of which six were based on fossils [20] and one was a secondary calibration point taken from a previous publication [14]. All calibration priors were modeled as normal distributions with a mean and a wide standard deviation (Table 1), as such soft priors take into account the bidirectional error associated with the use of fossil calibrations [21], [17].

**Table 1:** Cailbration points used to estimate times of divergence in Lepidoptera. Taxa from Mutanen *et al*. (2010) that were used to define the calibrated nodes are given in parentheses.

Calibration	Type	Calibration age (mya ± S.D.)	Source
Root	fossil (*Archaeolepis mane*)	190±10	[6]
Nepticuloidea (*Ectoedemia, Opostega*)	fossil (leaf mines)	120±10	[22]
Incurvarioidea (*Incurvaria, Prodoxus*)	fossil (unnamed)	120±10	[23]
gelechioids (*Anatiplora, Oecophora*)	fossil (unnamed)	120±10	[24]
Phyllocnistids (*Phyllocnistis, Phllonorycter*)	fossil (leaf mines)	99±10	[22]
bucculatricids (*Bucculatrix, Tritymba*)	fossil (*Bucculatrix platani*)	93±10	[25]
Nymphalidae (*Libythea, Nymphalis*)	secondary calibration point	90±5	[14]

The fossil calibrations were as follows. *Archaeolepis mane* from the Early Jurassic, which is thought to be the oldest fossil that can be confidently assigned to Lepidoptera [6], was used to calibrate the tree root (Table 1). Fossil leaf mines assigned to the family Nepticulidae from the Early Cretaceous were used to calibrate the split between *Ectoedemia* and *Opostega* [22]. The age of the crown clade of Incurvarioidea was calibrated using an unnamed fossil from the Early Cretaceous [23] assigned to the family Incurvariidae. Another Early Cretaceous fossil assigned to Gelechiidae [24] was used to calibrate the age of the crown clade of the superfamily Gelechioidea. Fossil leaf mines assigned to the gracillarid subfamily Phyllocnistinae from the Late Cretaceous [22] were used to calibrate the age of this subfamily. The fossil *Bucculatrix platani* from the Late Cretaceous [25] was used to calibrate the split between *Bucculatrix* and its sister group. The calibrated nodes (Table 1) were chosen based on the morphological interpretations of the authors in their publications of the fossil descriptions. Finally, we used the result from Wahlberg *et al*. [14] as a secondary calibration point to calibrate the age of the first split in Nymphalidae at 90 mya. The robustness of the resulting age estimates was assessed by removing each calibration sequentially but keeping the remaining six calibrations. The posterior densities of age estimates of all calibrated nodes were then visually assessed for the effects of the removal of each calibration on the age estimates for that node as well as on all the remaining calibrated nodes.

Four independent runs of 20 million generations, sampling every 2000 generations, were run using the program BEAST v1.7 [19]. The maximum likelihood topology from Mutanen *et al*. [8] (their Figure 1) was used as input and the four operators dealing with topology (subtreeSlide, narrowExchange, wideExchange and wilsonBalding) were turned off using BEAUti, thus the topology of the tree was fixed. Branch lengths were allowed to vary under a lognormal relaxed clock model and the tree prior was set to the birth-death model. The data were analyzed as one block with the GTR+G model assigned. The data was also analyzed as partitioned by gene, but the resulting output files were too large to be subsequently summarized on any computer available to us using TreeAnnotator (part of the BEAST package). The log and tree files were combined using LogCombiner (part of the BEAST package) with the first 3 million generations discarded as burnin from each run. We used Tracer v1.5 to assess whether the likelihood traces of the four runs had converged to a stable equilibrium and that ESS values were above 200 for all parameters.

**Figure 1:**
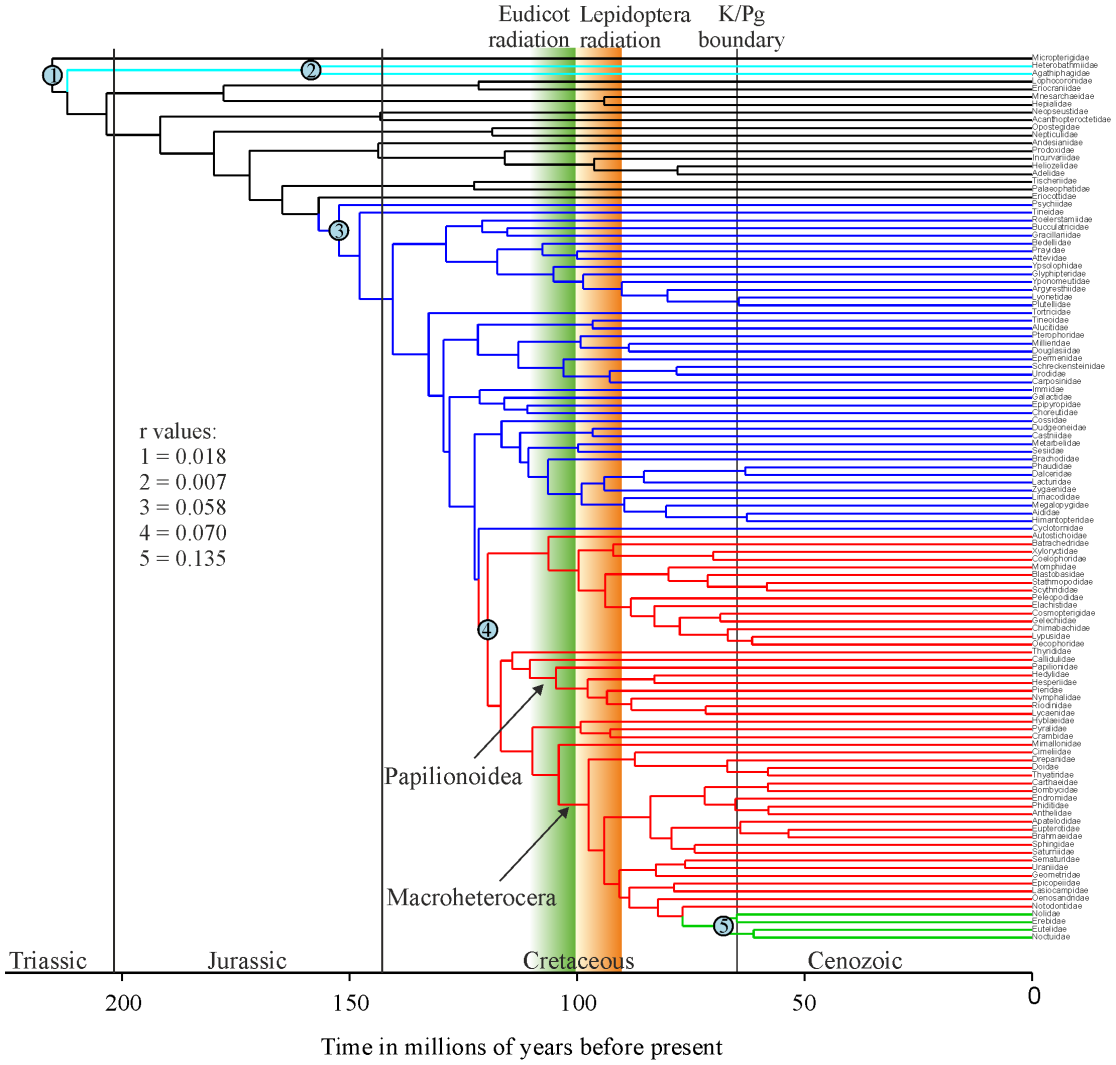
Estimated times of divergence for lineages leading to families of Lepidoptera based on 350 taxa (the full tree on which all analyses were performed is shown in Fig. S1). Changes in rates of lineage diversification based on a MEDUSA analysis (numbered nodes, see text for details) are shown on the topology with different colours corresponding to the *r* values. Time span for major eudicot radiations (according to [36]) is shown in green, and our estimated time span for a radiation in Lepidoptera based on our Δγ analysis (see Fig. 3) is shown in orange.

Diversification analysis used the algorithm Modeling Evolutionary Diversity Using Stepwise AIC (MEDUSA) [26] with species richness data for families in Lepidoptera [2]. The phylogenetic hypothesis of Mutanen *et al*. [8] included 116 out of 134 families based on the recently revised classification of Lepidoptera [2]. Missing families are all very species poor (<20 described species) and most are likely to be closely related to sampled families. Thus their exclusion is not expected to affect the broad results reported here. MEDUSA fits birth-death likelihood models on trees using increasing numbers of significant acceleration and deceleration breaking points based on branch lengths and species richness. The best model is selected based on AIC values, which includes values for net diversification rates and turnover.

In order to detect sudden increases in diversification of independent clades during the same period of time, the Δγ method [27] was used. This method extends the gamma statistics of Pybus and Harvey [28] to measure the increase or decrease of splits in a phylogenetic tree over user defined time intervals. The Δγ method was applied to 1000 trees sampled randomly from the post-burnin BEAST run in order to inspect the significance level of the diversification estimations. Phylogenetic trees were analyzed for several interval.width values (from 2 to 5 million years) using the code of McInnes *et al*. [27] (code available at https://github.com/carlosp420/deltagamma) in the statistical software package R [29]. Density plots were generated to observe the significant bursts of diversification obtained from all 1000 trees for each run using different interval.width values.

One of the open questions facing analyses of historical diversification rates is the potential bias arising from limited datasets, which could potentially lead to spuriously shorter branches within specific regions of a reconstructed topology. We explored this potential by assessing how a molecular dataset half vs. twice the size of the observed data would affect our conclusions. Analysis of the half-sized dataset was performed on ten datasets, each generated by randomly sampling without replacement from the observed dataset using the delete-half-jackknife option with 50% sampling, implemented via SEQBOOT from the PHYLIP v3.69 software package [30]. Upon each of these ten datasets and a simple doubling of the dataset, we repeated the Δγ analyses as described above.

## Results

Our results suggest that extant lineages of Lepidoptera began diversifying in the Late Triassic some 215 mya (Fig. 1). The nonditrysian lineages appear to have diverged during the early to mid Jurassic, and the first divergences in the huge clade Ditrysia happening in the late Jurassic. The main bulk of Ditrysia, beginning with the split of the yponomeutoid-gracillarioid clade from its sister group, appear to have diverged throughout the Cretaceous. Almost all lineages leading to extant families diverged from each other prior to the Cretaceous/Paleogene (K/Pg) event 65 mya, with the remaining lineages diverging soon after the event (Fig. 1). Extant diversity within families has quite likely evolved in the Cenozoic (Fig. S1). Interestingly, the huge clade Macroheterocera, which includes the megadiverse superfamilies Noctuoidea and Geometroidea, appears to be approximately the same age as the butterflies, ie. about 100 million years. Ages of crown clades in selected species rich superfamilies are given in Table 2.

**Table 2:** Ages in millions of years of selected superfamilies.

Superfamily	Age of crown	Range
Gracillarioidea	120	108–133
Yponomeutoidea	117	103–131
Tortricoidea	68	52–86
Gelechioidea	106	95–117
Papilionoidea	104	95–114
Pyraloidea	93	80–105
Bombycoidea	84	74–93
Geometroidea	83	72–93
Noctuoidea	82	73–92

**Figure 2:**
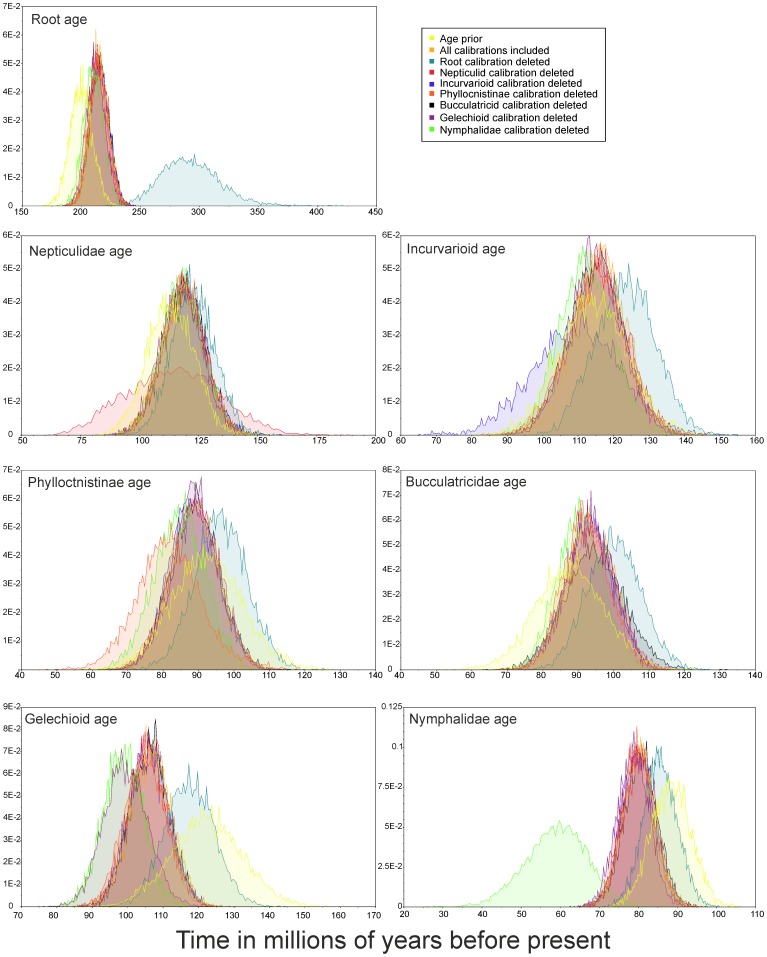
Posterior densities for age estimates for the calibrated nodes based on the full analysis (light orange distribution) as well as the sequentially removed calibrations (see inset for key to colors), also the prior distribution is shown (in yellow).

Removing each calibration sequentially did not have a large effect on the age estimate of the node for which the calibration was removed, nor for the other calibrated nodes in most cases (Fig. 2). The exception is the root calibration, the removal of which caused the age of the Lepidoptera crown clade to become much older (ca. 290 million years with a very wide credibility interval). However, the other calibrated nodes (for which the calibrations were retained) are estimated to be only marginally older than in the full analysis (light blue distributions in Fig. 2). Removal of the secondary calibration point for Nymphalidae causes the age of that clade to become substantially younger (ca. 60 million years), but again, the other calibrated nodes are not affected by this removal.

The MEDUSA method found three significant increases in diversification rates and one significant decrease along the maximum credibility tree of Lepidoptera (Fig. 1). The background diversification rate is estimated to have been 0.018 lineages per million years (*r*) and increases in the rates are associated with major clades of Lepidoptera. MEDUSA inferred a more than doubling of the diversification rate (*r* = 0.058) at the base of the clade Ditrysia around 150 mya (node 3 in Fig. 1), followed by another increase (*r* = 0.070) at the base of the clade Apoditrysia around 120 mya (node 4 in Fig. 1). The so-called quadrifid noctuoid families (Noctuidae, Eutelidae, Nolidae and Erebidae, see [31]) appear to have gone through a spectacular increase in diversification rates (*r* = 0.135) just at the end of the Cretaceous (node 5 in Fig. 1). The only significant decrease in diversification rates (*r* = 0.007) is found on the lineage leading to Heterobathmiidae and Agathiphagidae (node 2 in Fig. 1), which together only have 5 extant species.

**Figure 3:**
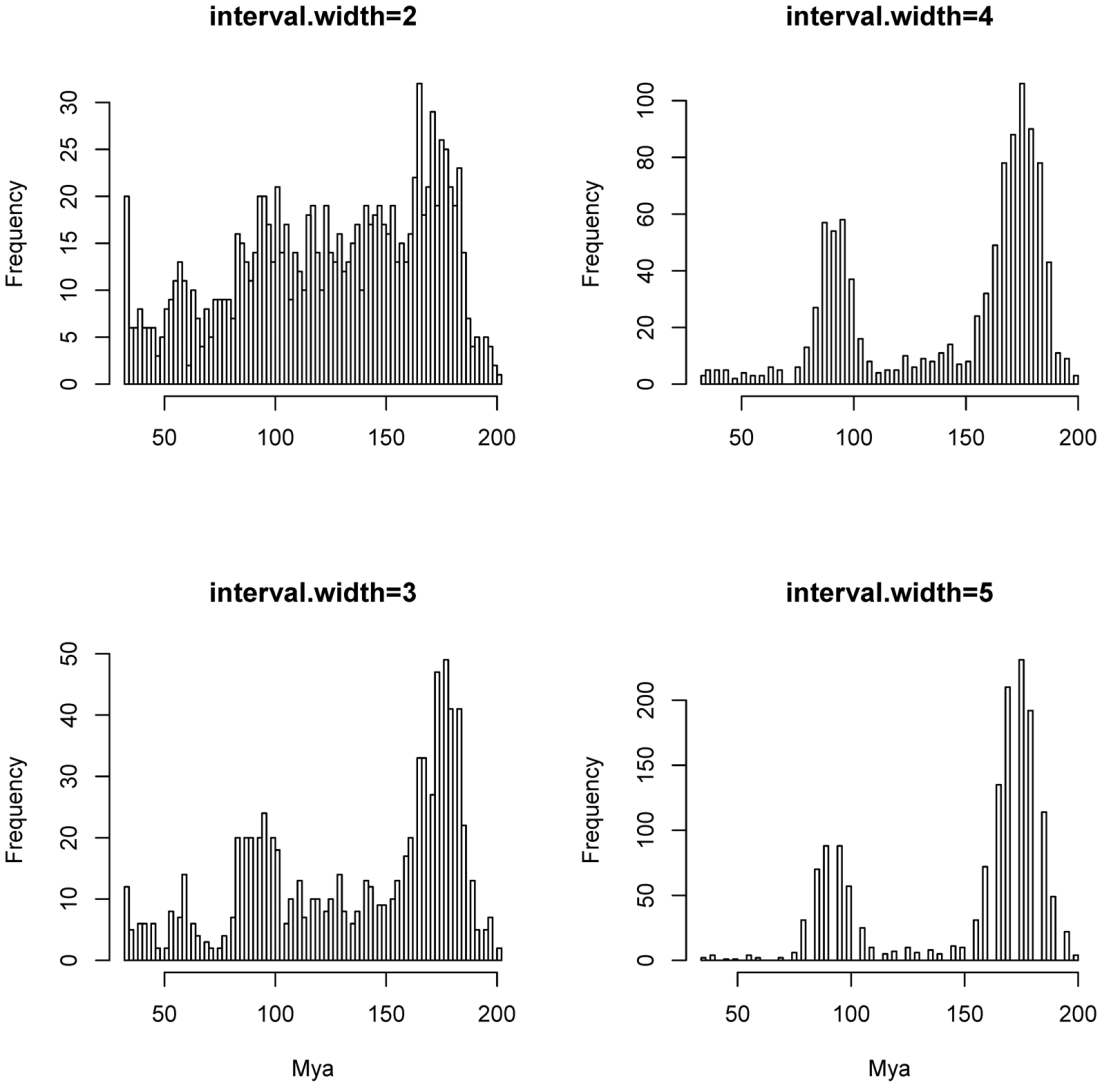
Histograms of frequency of significant diversification bursts estimated by Δγ on 1000 trees from the posterior distribution of the Bayesian run. The analyses were run with interval.width values from 2 to 5 million years.

Histograms of the diversification bursts found by the Δγ method on the posterior distribution of trees show clear patterns for analyses using the parameter “interval.width” with values from 2 to 5 million years (Fig. 3). These patterns suggest that significant diversification bursts happened at around 175 mya and at about 90 mya. The former may simply be a common artefact of methods that tend to show an early burst of diversification in large phylogenies even when the trees are generated by simulations using constant rate speciation and extinction parameters [32]. The latter diversification burst (at ca. 90 mya) suggests that Lepidoptera underwent a radiation in the Late Cretaceous. Analysis of the effect of dataset size on the peak at ca. 90 mya suggests that this finding is robust, as results from analyses on datasets one half and two times the size of the observed data all show the same diversification burst (at 90 mya) (Fig. S2).

## Discussion

According to our results, the extant diversity of Lepidoptera has evolved since the end of the Triassic, with the Jurassic being an important period for the diversification of the major nonditrysian lineages and the Cretaceous being the period of time when all major ditrysian lineages diverged from each other. These estimates are significantly older than those of Grimaldi & Engel [11], who suggest that both nonditrysian and the major ditrysian divergences happened in the Cretaceous. As a consequence, most of our estimated ages for crown clades of ditrysian superfamilies are also older than has previously been thought. Some of our results are explained by the differences in topologies, such as butterflies (Papilionoidea) not being nested within Macrolepidoptera, which in effect allows them to be older than the other clades in what is now called Macroheterocera. Other results are driven by the data and our calibrated nodes (of which three of the fossils were also used by Grimaldi & Engel [11]). In sum, our age estimates are generally 30–40 million years older than have previously been reported.

The accuracy and insights provided by age estimates are contingent on the calibrations used. In the ideal case, one would include morphological information from the fossils as well as extant taxa and analyze them together [33]. Unfortunately, at the time of writing this article, a comprehensive morphological dataset is not available for all Lepidoptera that would be informative with the inclusion of fossil data. Thus, the placements of the fossil taxa on the phylogenetic hypothesis used in this study are largely based on the extensive experience of the authors of the fossil descriptions. The exception is the fossil used to calibrate the crown clade of Lepidoptera, *Archeolepis*, which cannot be assigned to an extant lepidopteran family and is thought to represent a stem lepidopteran [6]. Such a fossil informs us that by 190 mya the lineages leading to Lepidoptera and Trichoptera had diverged from each other, but it does not inform us of whether the crown Lepidoptera had begun to diversify before or after that time period. We have used this fossil to inform our analysis that the age of the first split in Lepidoptera may be older or younger than 190 mya (a normal prior distribution around the calibrated node), depending on the data and the other calibrated nodes. The data and the combination of calibrated nodes used indicate that age of the crown clade of Lepidoptera is somewhat older (215 mya, 95% credibility interval 200–231 mya) than the fossil. Whether this is really the case remains to be investigated with a dataset that includes Trichoptera, but our analysis without the root calibration tended to make Lepidoptera much older (Fig. 2). Ages of the later divergences, from the early Cretaceous onwards, appear to be less affected by the removal of each calibration, suggesting that our inferences regarding the diversification of Lepidoptera with regard to the diversification of angiosperms are robust.

A few molecular studies have results that can be compared with our findings. A study investigating the relationships and times of divergence of holometabolous insect orders [34] found that Lepidoptera diverged from its sister group Trichoptera in the Triassic some 230 mya. This is highly consistent with a recent study on the evolutionary history of Trichoptera, which estimated that the trichopteran and lepidopteran lineages diverged 234 mya [12]. The recent phylogenomic analysis of arthropod times of divergences [17], which included 15 lepidopteran species and no fossil constraints for that order, estimated somewhat older times of divergence than our current estimates, although the credibility intervals overlap considerably. For instance, the most common recent ancestor of the nonditrysian Prodoxidae and the ditrysians was estimated to have been around approximately 198 mya (95% credibility interval 171–225 mya), whereas here we estimate it to have been later at 172 mya (95% credibility interval 158–186 mya). The older estimates of Wheat and Wahlberg [17] might be a result of low taxon sampling within Lepidoptera as well as missing holometabolan lineages, such as Trichoptera. On the whole, the molecular results tend to be consistent with the hypothesis that Lepidoptera are older than their poor fossil record suggests.

Lepidoptera appear to be as old as the angiosperms, which are also thought to have diverged from their sister group in the Triassic [35], [36]. If this is the case, Lepidoptera have had the possibility to interact with angiosperms throughout their evolutionary history. Larvae of early lineages are associated with woody angiosperms (e.g. Heterobathmiidae, Eriocraniidae, Nepticulidae and Opostegidae), but also with bryophytes and detritus (Micropterigidae and Mnesarchaeidae), gymnosperms (Agathiphagidae) and generalist root feeders (Hepialoidea) [3]. Further up the tree, larval associations with woody and herbaceous angiosperms becomes widespread, until herbaceous angiosperms dominate in ditrysian lineages. Based on these patterns, it can be hypothesized that ancestral Lepidoptera were associating with ancestral angiosperms as well as other available plants (bryophytes, gymnosperms) soon after the lepidopterans diverged from the common ancestor with trichopterans (which are mainly detrivores and carnivores). As angiosperms became more important components of ecosystems, lepidopteran lineages able to exploit them diversified, with the first increase in diversification rates some 150 mya (Fig. 1), which is coincident with the first divergences in the Mesangiospermae [35]. Continued association with herbaceous angiosperms led to a second burst of diversification among the lepidopteran lineages soon after the radiation of such important host plant clades as rosids and asterids about 90 mya (Figs. 1 and 2).

Support for phylogenetic relationships of major lineages in Lepidoptera is notoriously low [7]–[9] and it might be construed that this would have a large effect on our current results. However, our results are robust to the exact relationships as the chosen calibration nodes are well-supported and the diversification analyses are not dependent on the order of dichotomous divergences, as long as they have happened within approximately the same slice of time. Furthermore, we find that even when using only half our data, the burst of diversification is still observed (Fig. S2). We thus feel that our inferences of Jurassic divergences of basal lepidopteran lineages and Cretaceous divergences of the major ditrysian lineages, as well as our inference of a burst of lepidopteran diversification at about 90 mya, are robust.

Based on our results, divergences leading to extant lepidopteran families have happened almost exclusively in the Cretaceous (Fig. 1), but diversification within families has mainly happened in the Cenozoic (Fig. S1). This pattern has been proposed for butterflies earlier [14], [16], where it was suggested to be the result of widespread extinctions caused by the K/Pg event. More intensive sampling of the species rich lepidopteran families would be necessary to investigate whether the K/Pg event had an even broader impact on Lepidoptera diversity. Considering the extinction events that followed the rise of the Ditrysia over 100 million years ago, that we are able to identify any hints of ancient diversification events is impressive, for “… we view only the remnants, doubtless often disarranged if not completely shattered by subsequent events, of the great adaptive radiations of the past” [4] (pg 604).

In conclusion, we are able to give an evolutionary timeframe for the order Lepidoptera for the first time based on fossil-calibrated analyses with relaxed-clock methods. Our estimated times of divergence are older than previous estimates based on intuitive appraisal of fossils on phylogenetic hypotheses, yet appear to be corroborated by several independent studies focussed on other taxa. Our study suggests that diversification of lepidopteran lineages happened concurrently with diversification of angiosperm lineages, suggesting that co-evolutionary processes have had a chance to act over the entire evolutionary history of both the angiosperms and their insect herbivores. In addition, it appears clear that the K/Pg event set the stage for the diversification of the lineages that have led to the crown clades of extant families.

## Supporting Information

Figure S1 **The full 350 taxon timed tree of Lepidoptera families upon which all analyses were performed.** The tree includes 95% credibility intervals for the age estimates of each node.
(PDF)

Figure S2 **Histograms of frequency of significant diversification bursts estimated by Δγ on 1000 trees from the posterior distribution of Bayesian runs on 10 randomly halved datasets (see text for details).**
(PDF)
