# Supplementary figures and images for: Timing and Patterns in the Taxonomic Diversification of Lepidoptera (Butterflies and Moths)
Source: PLoS One. 2013 Nov 25;8(11):e80875. doi: 10.1371/journal.pone.0080875 (PMC3839996; doi:10.1371/journal.pone.0080875)

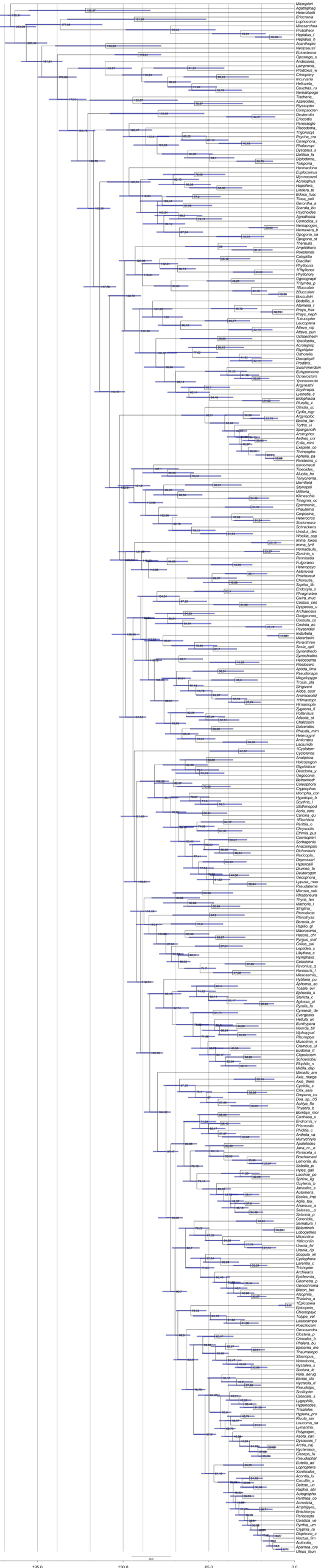

Supplement: Figure S1 — The full 350 taxon timed tree of Lepidoptera families upon which all analyses were performed. The tree includes 95% credibility intervals for the age estimates of each node. (PDF) [file pone.0080875.s001.pdf]

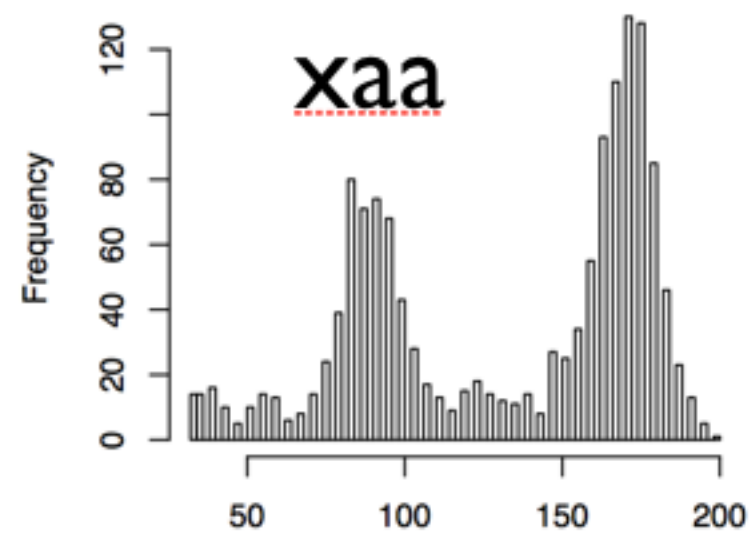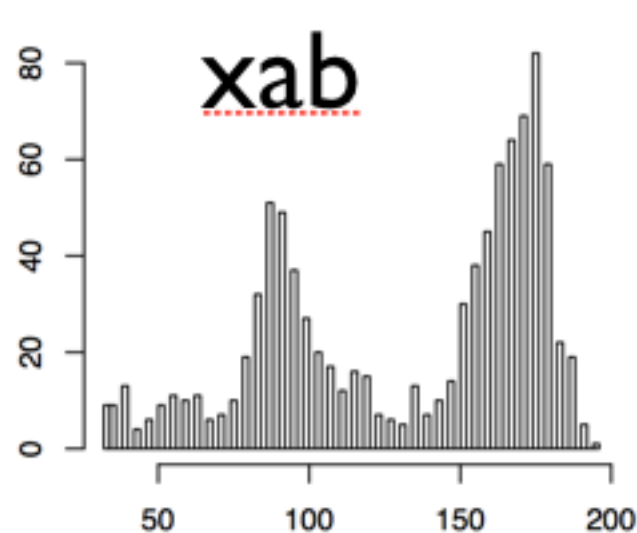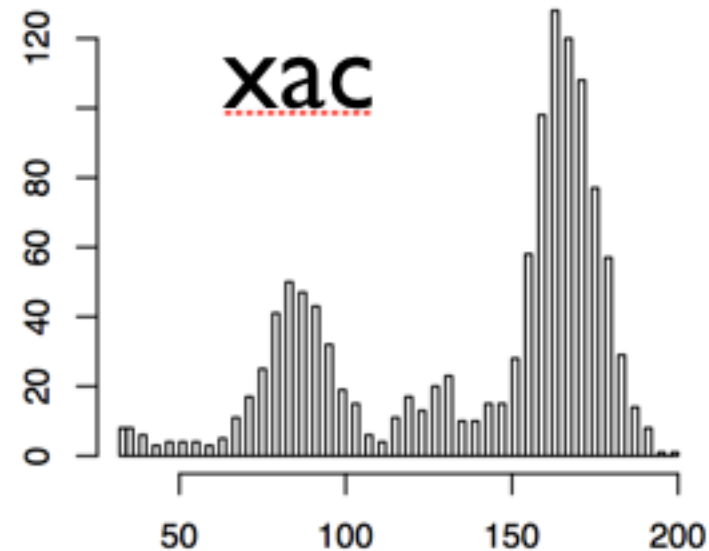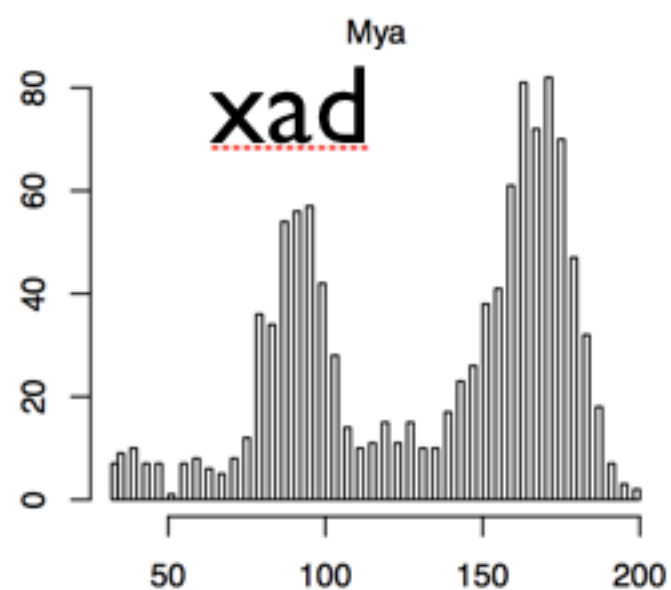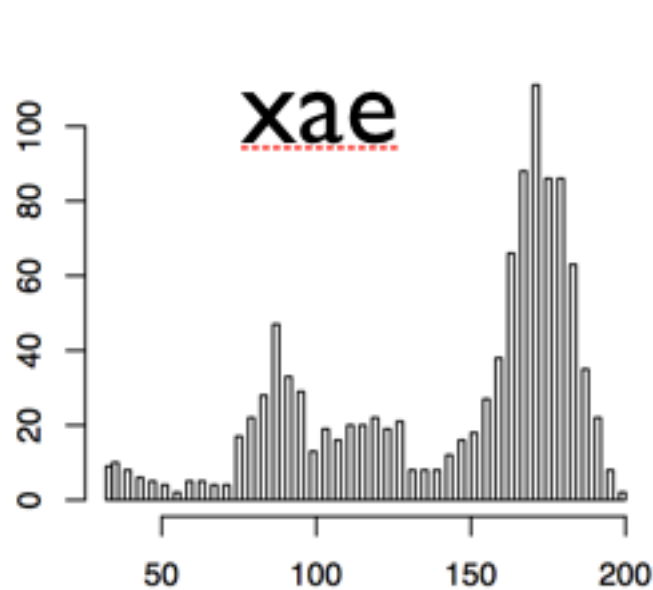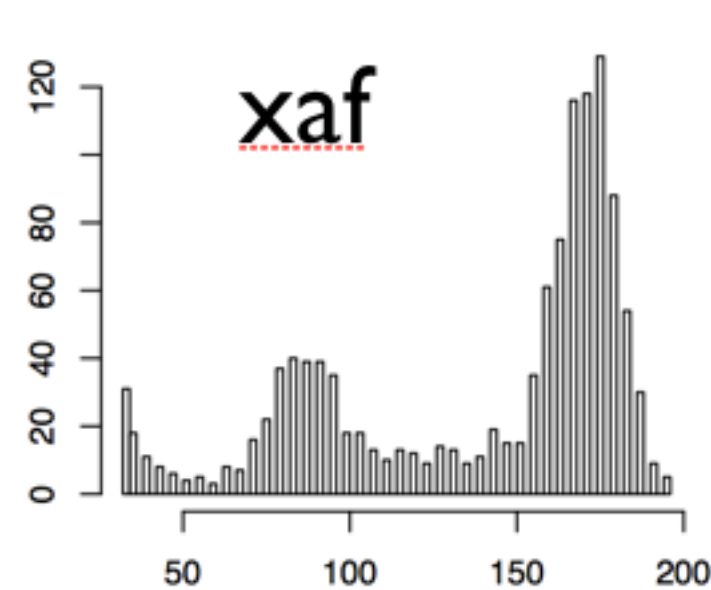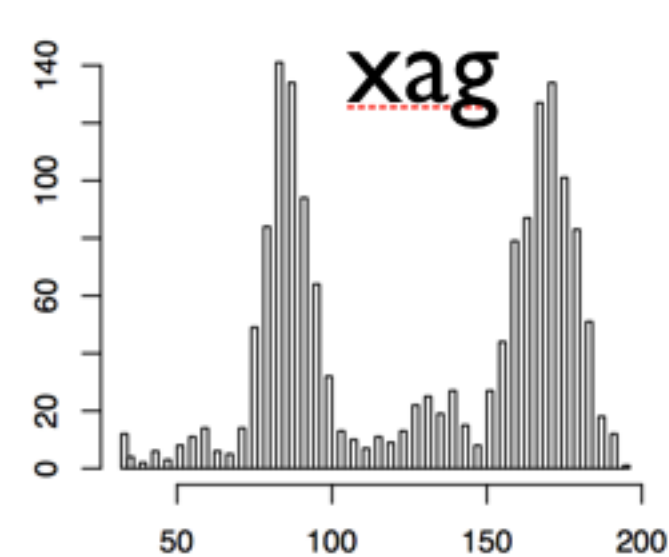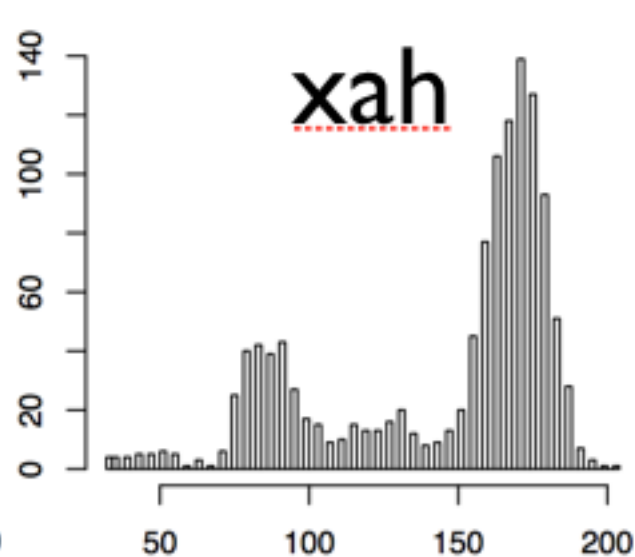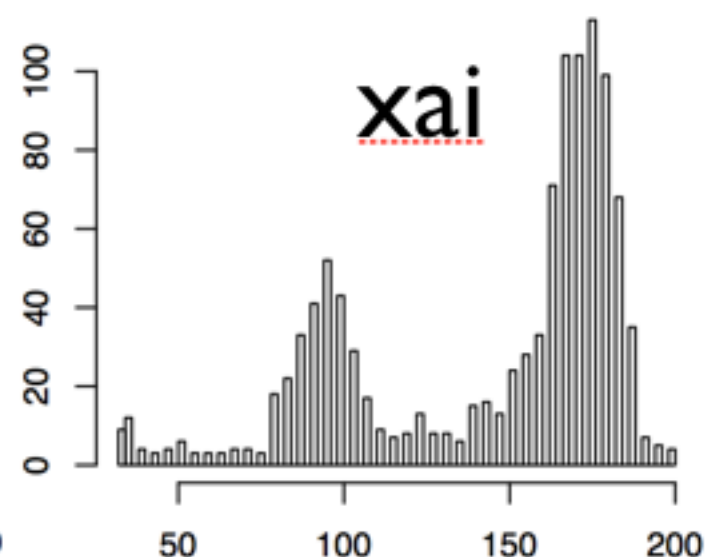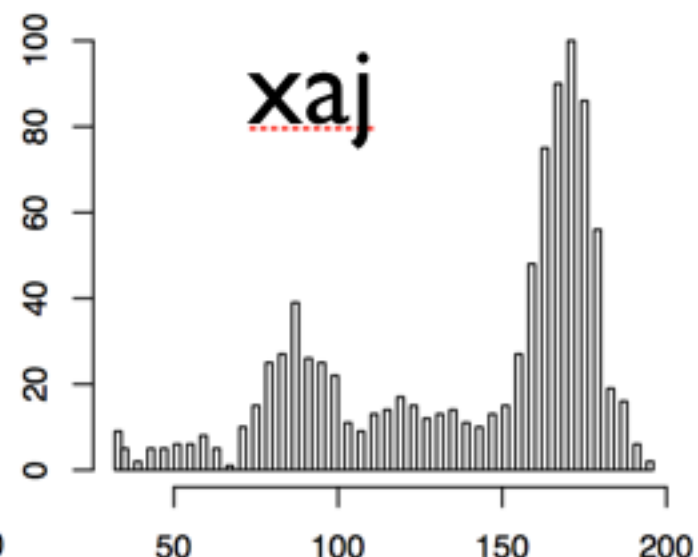

Supplement: Figure S2 — Histograms of frequency of significant diversification bursts estimated by Δγ on 1000 trees from the posterior distribution of Bayesian runs on 10 randomly halved datasets (see text for details). (PDF) [file pone.0080875.s002.pdf]
